# Examining joint attention with the use of humanoid robots-A new approach to study fundamental mechanisms of social cognition

**DOI:** 10.3758/s13423-019-01689-4

**Published:** 2019-12-17

**Authors:** Pauline Chevalier, Kyveli Kompatsiari, Francesca Ciardo, Agnieszka Wykowska

**Affiliations:** Social Cognition in Human–Robot Interaction Unit, Istituto Italiano di Tecnologia, Genoa, Italy

**Keywords:** Joint attention, Human–robot interaction, Healthy and clinical populations, Autism, Review

## Abstract

This article reviews methods to investigate joint attention and highlights the benefits of new methodological approaches that make use of the most recent technological developments, such as humanoid robots for studying social cognition. After reviewing classical approaches that address joint attention mechanisms with the use of controlled screen-based stimuli, we describe recent accounts that have proposed the need for more natural and interactive experimental protocols. Although the recent approaches allow for more ecological validity, they often face the challenges of experimental control in more natural social interaction protocols. In this context, we propose that the use of humanoid robots in interactive protocols is a particularly promising avenue for targeting the mechanisms of joint attention. Using humanoid robots to interact with humans in naturalistic experimental setups has the advantage of both excellent experimental control and ecological validity. In clinical applications, it offers new techniques for both diagnosis and therapy, especially for children with autism spectrum disorder. The review concludes with indications for future research, in the domains of healthcare applications and human–robot interaction in general.

## Introduction

In this review, we describe a novel approach for studying the mechanisms of joint attention, namely the use of robot agents as dynamic “social stimuli” in naturalistic interactive scenarios. We argue that such a method provides more ecological validity than do classical screen-based protocols, while simultaneously allowing excellent experimental control. After a brief review of classical studies on joint attention, and the more recent approaches, we focus on the approach of using embodied robots in interactive scenarios. In the final section, we describe application areas in which robots are used to train joint attention skills in children diagnosed with autism spectrum disorder (ASD). Using robots for examining joint attention (and social cognition in general) is very timely, due to the recent emergence of new approaches in the study of human social cognition, the so-called “Second-person Neuroscience” (Schilbach et al., 2013), new developments in clinical applications (Pennisi et al., 2016), and a current strong focus of academia, industry and society on artificial intelligence, robotics, human–robot interaction and the societal, as well as economical, impact of new digital technologies (Manyika et al., 2013).

### Classical studies on joint attention

Joint attention, a fundamental mechanism of social cognition (Frischen, Bayliss, & Tipper, 2007; Jording, Hartz, Bente, Schulte-Rüther, & Vogeley, 2018), has been widely studied in laboratory settings with the use of screen-based tasks. Joint attention is observed as the phenomenon of attending toward the same direction, or toward the same object/event, that another person is attending (Emery, 2000). The ability to discriminate between straight and averted gaze appears early in development (i.e., among 2-day-old babies—Farroni, Csibra, Simion, & Johnson, 2002; see also Vecera & Johnson, 1995) and it is considered a valid predictor of efficient development in linguistic abilities (e.g., Brooks & Meltzoff, 2005).

**Fig. 1:**
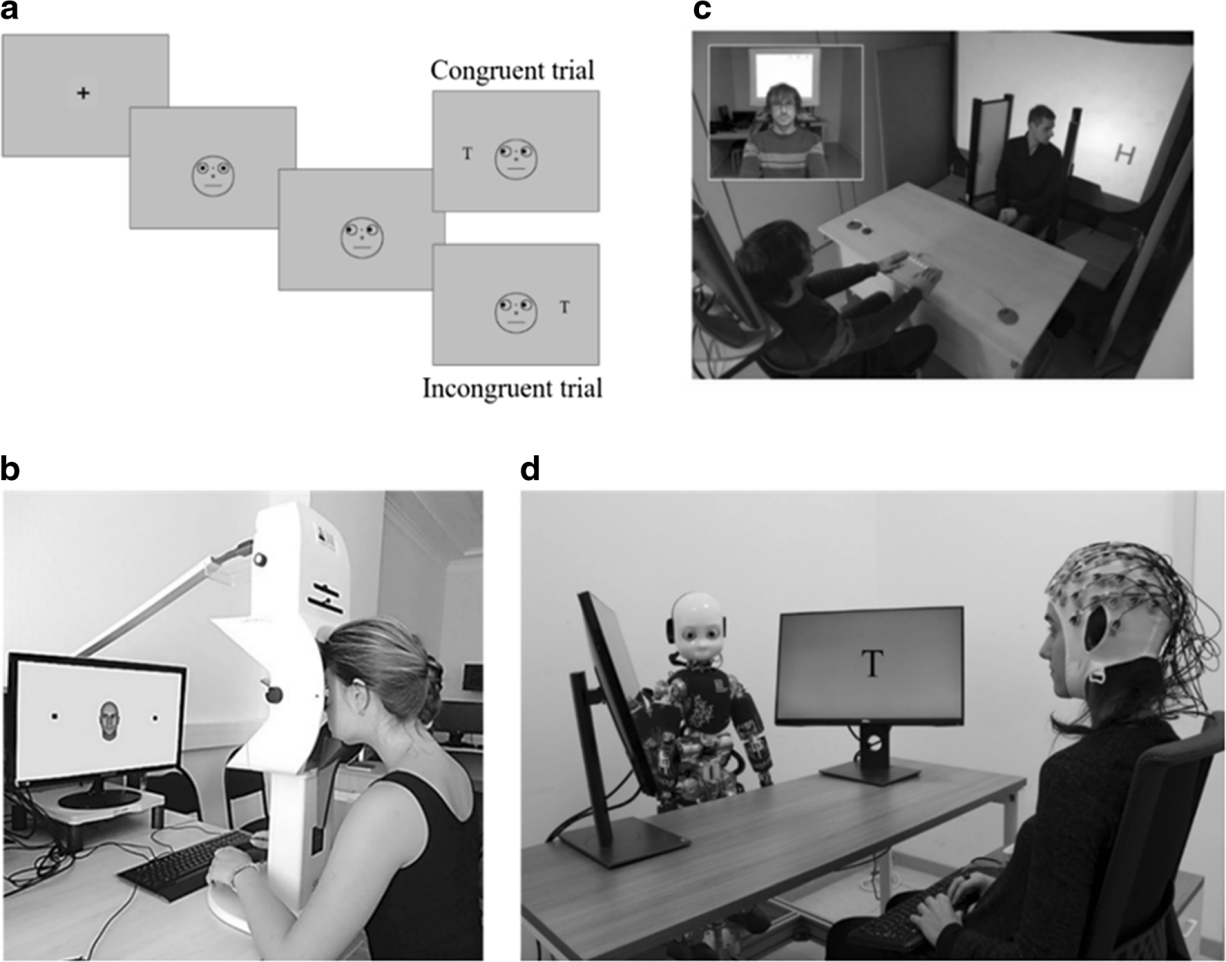
Examples of classical and novel paradigms used to study joint attention. (a) A gaze-cueing paradigm with schematic faces for congruent (upper frame) and incongruent (lower frame) trials (Friesen & Kingstone, 1998). From Ciardo et al., 2018. (b) Experimental setup in a gaze-following task using avatar faces. From “Studying the Influence of Race on the Gaze Cueing Effect Using Eye Tracking Method,” by G. Y. Menshikova, A. I. Kovalev, and E. G. Luniakova, 2017, *National Psychological Journal*, *2*, p. 50, Fig. 1. Copyright 2017 by Lomonosov Moscow State University and the Russian Psychological Society (Menshikova, Kovalev, & Luniakova, 2017). (c) Adapted gaze-cueing procedure for gaze cueing in a real-world experimental setup. From “Mental State Attribution and the Gaze Cueing Effect,” by G. G. Cole, D. T. Smith, and M. A. Atkinson, 2015, *Attention, Perception, & Psychophysics*, *77*, Fig. 5. Copyright 2015 by the Psychonomic Society (Cole, Smith, & Atkinson, 2015). (d) Gaze-cueing task in human–robot interaction (paradigm of Kompatsiari, Ciardo, et al., 2018).

In the last 20 years, joint attention has been studied by using pictures or schematic faces presented to participants on a computer screen, and it is often operationalized as a modification of Posner’s cueing paradigm (Posner, 1980): the *gaze-cueing paradigm.* In a typical experimental condition, represented in Figs. 1a and 1b, participants view a schematic or realistic picture of a face presented in the center of the display. The first image is then replaced with the same image with eyes averted to the left or to the right (i.e., gaze cue). Finally, a target may appear in the location signaled by the eyes (i.e., validly cued trials) or in the opposite location (i.e., invalidly cued trials). The averted gaze represents the cue while its predictivity regarding target location is usually one of the variables that are manipulated in such paradigms. As in the classic spatial-cueing paradigm, responses are faster for validly than for invalidly cued trials (i.e., gaze-cueing effect), indicating that attention is oriented in the direction signaled by the gaze and thus switching focus to the uncued location is costly. One of the first studies investigating this phenomenon was carried out by Friesen and Kingstone (Friesen and Kingstone,^1998^; see also Driver et al., 1999). Electrophysiological and neuropsychological evidence highlighted the relationship between gaze direction and attention, indicating the existence of a specific neural substrate devoted to process meaningful gaze direction (i.e., gaze directed toward an object rather than toward empty space), like the superior temporal sulcus (STS; Allison, Puce, & McCarthy, 2000; Hoffman & Haxby, 2000; Pelphrey, Singerman, Allison, & McCarthy, 2003; Perrett et al., 1985). The STS projects input–output connections from- and to the fronto-parietal attentional networks (Corbetta, Miezin, Shulman, & Petersen, 1993; Maurizio & Shulman, 2002; Harries & Perrett, 1991; Nobre et al., 1997; Rafal, 1996). Through these connections, information about gaze direction projects to spatial attention systems to orient attention in the corresponding direction, as it occurs in joint attention.

### Bottom-up and top-down components in joint attention

Early behavioral and electrophysiological studies investigating the gaze-cueing effect showed that the orienting of attention triggered by averted gaze can be defined as automatic (Jonides, 1981). Indeed, it has been showed that gaze-cueing effect emerges early in time (Friesen & Kingstone, 1998; Frischen et al., 2007), and is not affected by the nature of the task (Friesen & Kingstone, 1998), by gaze predictivity (Driver et al., 1999), or by a secondary, resource-demanding task (i.e., a memory task; Law, Langton, & Logie, 2010). Event-related potentials (ERPs) showed that occipital–parietal P1 and N1 components are modulated by gaze validity, indicating that visual processing already in the extrastriate cortex is modulated by gaze cues (Perez-Osorio, Müller, & Wykowska, 2017; Schuller & Rossion, 2001). Furthermore, Ricciardelli, Bricolo, Aglioti, and Chelazzi (2002) developed a prosaccade/antisaccade task to investigate whether observed averted gaze can interfere with goal-driven saccades (i.e., the *gaze-following paradigm*; see also Ciardo, Marino, Actis-Grosso, Rossetti, & Ricciardelli, 2014; Ciardo, Marino, Rossetti, Actis-Grosso, & Ricciardelli, 2013; Ricciardelli, Carcagno, Vallar, & Bricolo, 2013, for results using the same paradigm). Saccadic performance is less accurate when the gaze cue is incongruent with the saccade instruction. Recent studies, however, suggest that joint attention may not be purely bottom-up driven, but it is rather a combination of bottom-up and top-down mechanisms. Several factors have been identified to have an impact on top-down modulation of the gaze-cueing effect: relevance for the task (e.g., Ricciardelli et al., 2013), other stimuli in the environment (e.g., Greene, Mooshagian, Kaplan, Zaidel, & Iacoboni, 2009; Ristic & Kingstone, 2005), whether the gazing agent is assumed to see the target (Teufel, Alexis, Clayton, & Davis, 2010), believed reliability of the gazing agent (Wiese, Wykowska, & Müller, 2014), or whether the gaze is in line with action expectations (Perez-Osorio, Müller, Wiese, & Wykowska, 2015; Perez-Osorio et al., 2017). Furthermore, also *social* information associated with the observed agent plays a role in gaze-cueing effect: age (e.g., Ciardo et al., 2014; Ciardo et al., 2013), social status (e.g., Ciardo et al., 2013; Dalmaso, Pavan, Castelli, & Galfano, 2012); social attitude (Carraro et al., 2017; Ciardo, Ricciardelli, Lugli, Rubichi, & Iani, 2015), or assumed intentionality (Wiese, Wykowska, Zwickel, & Müller, 2012; Wykowska, Wiese, Prosser, & Müller, 2014). Taken together, these results highlight a link between joint attention and other (higher-level) mechanisms of cognition (see Capozzi & Ristic, 2018, for review) suggesting that engagement in joint attention in everyday life may be dependent on contextual and social information.

### Joint attention, development, and individual differences

Gaze following behavior plays a pivotal role in development. For example, even children as young as 3 months are able to discriminate averted gaze and to shift attention to the corresponding location (Hood, Willen, & Driver, 1998). Moreover, longitudinal studies showed that an early onset of gaze-following predicts efficient development in linguistic abilities (e.g., Brooks & Meltzoff, 2005). Several studies showed that joint attention is dependent on individual differences, such as self-esteem (Wilkowski, Robinson, & Friesen, 2009), gender (Bayliss & Tipper, 2006), and autistic traits (Bayliss, di Pellegrino, & Tipper, 2005). For instance, Bayliss et al. (2005) reported a negative correlation between gaze-cueing effect magnitude and score on the Autism-Spectrum Quotient questionnaire (Baron-Cohen, Wheelwright, Skinner, Martin, & Clubley, 2001). Similarly, Ristic and Kingstone (2005) showed that adults diagnosed with high functioning autism show the gaze-cueing effect only when gaze direction is informative with respect to the possible location of the target, suggesting that for adults diagnosed with autism spectrum disorder gaze direction does not have the special status typically observed in healthy controls. A study investigating joint attention in patients suffering from chronic schizophrenia showed weaker gaze-cueing effect (Akiyama et al., 2008), whereas standard cueing effects were reported for non-social cues (i.e., arrows) and pointing gestures (Dalmaso, Galfano, Tarqui, Forti, & Castelli, 2013; see Marotta et al., 2014, for similar results from ADHD patients). Langdon & colleagues (2017) showed that when pictures of real faces instead of schematic faces are used, the larger gaze-cueing effect reported in schizophrenia patients can be attributed to a difficulty in disengaging from the gazed-at location once shared attention is established (Langdon, Seymour, Williams, & Ward, 2017). Altogether, these findings strongly support the idea that the ability to respond to joint attention signals and the development of communicative and social skills are strongly connected. However, classical studies use pictures or schematic faces presented to participants on a computer screen and mainly focus on responding to joint attention. Such classical paradigms contribute to understanding the cognitive and neural mechanisms of joint attention but lack the aspect of reciprocity in social interactions and ecological validity (Schilbach, 2015).

### Recent approaches to study joint attention, highlighting the need for reciprocity

Recently, a new framework has been proposed according to which studying mechanisms of social cognition require experimental paradigms involving more “online” social interaction (Bolis & Schilbach, 2018; Edwards, Stephenson, Dalmaso, & Bayliss, 2015; Kajopoulos, Cheng, Kise, Müller, & Wykowska, in press; Risko, Laidlaw, Freeth, Foulsham, & Kingstone, 2012; Risko, Richardson, & Kingstone, 2016; Schilbach, 2014; 2015; Schilbach et al., 2013).

There is evidence that findings from static stimuli used in traditional paradigms cannot evoke the same mechanisms of response to joint attention as more dynamic social stimuli (for a review, see Risko et al., 2012). To begin with, even though Hietanen and Leppänen (2003) using static gaze cues found a similar gaze-cueing effect across emotions (happy, sad, fearful), Putman and colleagues using more complex dynamic representation of emotion and gaze found that the gaze-cueing effect was modulated by the emotion—that is, larger cueing effect for fearful than for happy faces (Putman, Hermans, & van Honk, 2006). The modulation of emotion on gaze-cueing effect might be associated with the difference in emotion processing per se that seems to be enhanced using dynamic stimuli (Sato, Kochiyama, Yoshikawa, Naito, & Matsumura, 2004; Sato & Yoshikawa, 2007). Importantly, studies have also examined the classical gaze-cueing paradigm using another human as a central cue. For example, Cole, Smith, and Atkinson (2015) examined the effect of mental state attribution on gaze-cueing effect during a human–human interaction. They found robust gaze-cueing effect even when the person’s view was occluded from the targets (a mental state of “not seeing”; see Fig. 1c), which is in contrast with previous screen-based studies in which the gaze-cueing effect was modulated by the belief regarding whether the gazer can or cannot see through a pair of goggles (Teufel et al., 2010). Interestingly, Cole and colleagues found a gaze-cueing effect approximately three times larger than that for standard screen-based stimuli (see Lachat, Conty, Hugueville, & George, 2012, for a different pattern of results, when only eyes are used as a cue instead of the whole head movements).

The abovementioned studies provide evidence that using more dynamic and naturalistic social stimuli in joint attention research might lead to different findings than static, screen-based stimuli. This is further confirmed by several efforts that have been made to study mechanisms of joint attention in the “wild”—that is, in situations that involve or have the potential for real social interaction (for a review, see Risko et al., 2012). In this case, evidence suggests that results from laboratory paradigms are not necessarily valid in natural, real world situations. For example, Gallup and colleagues showed that participants were more likely to follow cues of confederates toward an attractive object when the confederates were walking in the same direction as them on the street (participants’ gaze direction could not be seen by the confederate), as compared to the opposite direction (participants’ gaze direction could be detected by the confederate) (Gallup, Chong, & Couzin, 2012a). Interestingly, when the “pedestrians” were facing them, participants not only did not follow their gaze, but they were also less likely to look at the attractive object compared to the baseline condition, in which no one had looked at the object before (see also Gallup et al., 2012b, for similar results). Hayward, Voorhies, Morris, Capozzi, and Ristic (2017) compared gaze following between a real-world interaction and a typical laboratory task. During real-world interaction, a confederate kept an everyday conversation with the participant, while maintaining eye contact, but shifted his/her gaze on five different occasions. Response to joint attention was operationalized as the proportion of the confederate’s gaze shifts that were followed by the participant. In the laboratory paradigm, participants executed a typical nonpredictive gaze-cueing task with a schematic face. In this task, response to joint attention was operationalized during the cue presentation period, as the proportion of trials in which participants broke fixation at the central cue and executed a saccade toward the gazed-at location. Additionally, the authors measured the traditional gaze-cueing effect as reflected by reaction times to target detection. Although results of attentional shifting were statistically reliable and consistent with the existing literature in both paradigms (real-world, laboratory), comparison between experiments showed that no reliable associations emerged for shifting functions between cueing task and real-world interactions. So far, studies “in the wild” show that findings collected in the laboratory do not necessarily reveal all factors playing a role in social cognition (for a review, see Risko et al., 2012).

The need for more naturalistic online social interaction protocols is even clearer with respect to the mechanism of initiating joint attention (rather than only responding to joint attention bids). Under this perspective, authors started using virtual agents in the experiments addressing the initiation of joint attention (Bayliss et al., 2013; Dalmaso, Edwards, & Bayliss, 2016; Edwards et al., 2015; Schilbach et al., 2009). Virtual agents can provide high levels of behavioral realism—for instance, in mimicking human eye movement capabilities with respect to appearance and timing (Admoni & Scassellati, 2017). To address the issue of reciprocity in social interaction—for example, gaze contingency—some studies involved an experimental setup with an interactive eye-tracking system monitoring participants’ gaze position on a stimulus screen and controlling gaze behavior of an anthropomorphic virtual character (Pfeiffer, Timmermans, Bente, Vogeley, & Schilbach, 2011; Schilbach et al., 2006; Wilms et al., 2010). By programming a virtual agent’s gaze behavior to be contingent on participant’s gaze, Schilbach et al. (2009) compared the neural correlates of joint attention in terms of initiating and responding to joint attention. Authors found that, whereas following someone else’s gaze activated the anterior portion of medial prefrontal cortex (MPFC), seeing someone else following our gaze direction also activated the ventral striatum, an area associated with different stages of reward processing, such as hedonistic and motivational aspects (Liu et al., 2007; Rolls, Grabenhorst, & Parris, 2008), highlighting thereby that reciprocity in joint attention has an impact on crucial engaging factors. Moreover, Redcay et al. (2010) developed an experimental setup that allowed the examination of face-to-face interactions between a participant inside an MRI scanner and an experimenter outside of the scanner through a real-time video feed of either live or previously recorded interaction (Redcay et al., 2010). The experimenter and the participant were engaged in a game in which they had a common goal to find a target (Redcay, Kleiner, & Saxe, 2012). In each trial, the participant either responded to joint attention by following the experimenter’s gaze to the target object (only the experimenter could see the clue about the location) or initiated joint attention by cueing the experimenter to look at the object (only the participant could see the clue about the location). In contrast to previous studies (Schilbach et al., 2009), this paradigm required the intentional coordination of attention toward a common goal. The study found that dorsomedial prefrontal cortex (dMPFC) was activated both in response to joint attention and initiating joint attention. However, initiating joint attention, specifically, recruited regions associated with attention orienting and cognitive control systems (see Caruana, McArthur, Woolgar, & Brock, 2017, for an extensive review on fMRI studies of joint attention).

At a behavioral level, Bayliss et al. (2013) developed a gaze-leading paradigm in which participants were asked to choose freely—by gaze direction—an object. A centrally presented face would either gaze at the same direction (gaze congruent) or at the opposite (gaze incongruent). After selecting the object, participants were required to look back to the central face (Bayliss et al., 2013). In line with the developmental importance of refocusing to our interaction partner (for a review, see Feinman, Roberts, Hsieh, Sawyer, & Swanson, 1992), the successfully initiated joint attention modulated the return-to-face saccades to the central face. More specifically, the return-to-face saccade onset times were slower when the gaze of the face was incongruent with participants’ gaze than in the congruent condition. Along a similar line, Edwards et al. (2015) showed that participants’ attention was shifted to peripherally presented faces who followed their gaze. Additionally, Dalmaso et al. (2016) showed that gaze-cueing effect was more prominent with faces who previously did not follow participants’ gaze, in comparison with faces who followed participants.

Taken together, these studies suggest that the two mechanisms of joint attention—that is, responding to joint attention and initiating joint attention—are not identical in nature, since they activate both common (MPFC) but also distinct brain areas considering that initiating joint attention specifically recruited areas related to reward processing, attentional orienting and cognitive control. Importantly, this shows that initiation of joint attention requires interactive protocols, and thus, classical “spectatorial” approaches with participants passively observing screen-based stimuli are not sufficient to elucidate the full plethora of mechanisms engaged in the mechanism of joint attention.

### Limitations of recent approaches to study joint attention

Studies using more ecologically valid experimental protocols suggest that findings in naturalistic setups might be different from screen-based “spectatorial” paradigms. Such interactive protocols have certainly advanced our knowledge regarding responding and initiating to joint attention, but each protocol involves specific shortcomings. For example, on the one hand, virtual agents can enable reciprocal social interactions but on the other hand, they still remain screen-based agents and thus lack the realism of natural social interactions. Human–human interaction paradigms increase the ecological validity but certainly impose challenges regarding the comparison between studies and the replicability of results, since various factors, such as the velocity of the directional movement during the cueing procedure, could influence the gaze-cueing effect in these setups. These factors are challenging to replicate, often they are not controlled for or not reported. Advancing to real-life paradigms poses even higher risk of compromising experimental control. For instance, apart from the controllability and reproducibility of the cues, differences in gazing arising from real-life situation, or from comparisons between live and screen-based cues can be attributed at least to some extent to the variations in the visual stimuli to which participants are exposed across situations (Gobel, Kim, & Richardson, 2015).

## Using robots to examine joint attention

Among the manifold recent approaches to examine human social cognition, there is a growing interest in using humanoid robot agents in joint attention studies. In more classical paradigms in which robot faces are presented on the screen, using such stimuli allows for answering the question of what is the role of humanness and human/natural agency in evoking joint attention mechanisms. That is, with artificial humanoid agents, we can examine whether human-likeness is a crucial factor for engagement in joint attention. In more interactive protocols with embodied humanoids, the advantage of using them is that they can overcome issues of recent interactive protocols by offering excellent experimental control on the one hand and allowing for increased ecological validity and social presence on the other. In this section, we will review studies that have used robot agents as attention-orienting stimuli in both screen-based as well as naturalistic protocols. Subsequently, we discuss possible limitations of using robots as interactive partners. In the final part of this section, we provide guidelines for optimal use of embodied humanoid robots in joint attention research.

### Screen-based paradigms examining joint attention with robot faces

The results from screen-based gaze-cueing paradigms with humanoid robots have not been entirely consistent. On the one hand, Admoni and colleagues found that two different robots, Zeno (Robokind) and Keepon, did not elicit reflexive gaze-cueing effect (Admoni, Bank, Tan, Toneva, & Scassellati, 2011). However, conclusions from this study are limited by the lack of statistical power (see Table 1), given the small number of cued trials (eight cued trials, p. 1986). In a similar line, Okumura, Kanakogi, Kanda, Ishiguro, and Itakura (2013) demonstrated that only a human gaze elicited anticipatory gaze shifts of 12-year-old infants, but robots did not have the same effect. On the other hand, Chaminade and Okka (2013) found that there was no difference in the magnitude of the gaze-cueing effects elicited by the head shift of a human face and of the NAO T14 robot face using nonpredictive cues (upper torso). Additionally, Wiese et al. (2012), by comparing the magnitude of gaze-cueing effect elicited by a robot and a human face using nonpredictive cues, demonstrated that both faces induced a gaze-cueing effect, but robots engaged participants in joint attention to a smaller extent. In a follow-up study, the authors showed that with the very same robot face, gaze-cueing effect was elicited, dependent on whether participants believed its behavior was preprogrammed or human-controlled (gaze-cueing effect was quantified both in reaction times and in the P1 component of the EEG signal). Martini, Buzzell, and Wiese (2015) studied the effect of the physical appearance of the robot (from 100% robot to 100% human) on mind attribution and gaze-cueing effect using a counter-predictive gaze-cueing paradigm. The authors found a positive linear relationship between mind attribution ratings and human-like appearance, however, this was not reflected in the gaze-cueing effect, which showed an inverted U-shaped pattern. Indeed, only agents with moderate level of human-likeness (60% human morph) induced automatic gaze-cueing effect, whereas both agents with 100% human-likeness (human faces) and 100% robot-likeness (robot faces) eliminated the gaze-cueing effect (Martini et al., 2015).

**Table 1:** Summary of the studies examining joint attention in healthy population, from classical to more naturalistic and recent approaches

Agent	Authors	*N*	SOA (ms)	GCE Magnitude (ms)	Effect Size (*d'*)
Screen based/Schematic and human faces	Friesen & Kingstone (1998)^*^	24	105, 300, 600, 1,005	7.5	1.11
	Schuller & Rossion (2001)	14	500	19	2.26
	Hietenan et al. (2006)	52	200	19	0.90
	Ciardo et al. (2018)^a^	32	200	16	2.58
	Dalmaso et al. (2016)^a^	19	200, 1200	19	1.97
Screen based/Avatars	Jones et al. (2010)^a^	20	200	10	0.49
	Pavan et al.(2011)^b^	32	200	12	1.14
Screen based/Robotic agent	Wiese et al. (2012)^a^ Wiese et al. (2012)^b^ Martini, Buzzell, & Wiese (2015)	23 46 35	500 500 400–600	9 9 7	1.96 1.71 0.77
Interactive setup/Human agent	Cole et al. (2015)^c^	16	600	n/a	2.94
	Lachat et al. (2012)	50	700–900	11	0.83
Interactive setup/Robotic agent	Wykowska et al. (2015)	34	600	13	1.32
	Kompatsiari, Perez-Osorio, et al. (2018)	21	500	15	0.73
	Kompatsiari et al. (2018)^a^	33	1,000	18	1.02

For each study we report the sample size (*N*); the stimulus onset asynchrony (SOA; separated by commas when multiple SOA were applied), the magnitude of the gaze-cueing effect (GCE; estimated as the difference in mean reaction times between invalid and valid trials; n/a = the authors did not report mean values for valid and invalid trials), and the effect size of the main validity effect (Cohen’s *d*, estimated using the Practical Meta-Analysis Effect Size Calculator), if calculable. ^*^We only report results of the identification task. ^a^ We only report results of Exp. 1. ^b^ We only report results of Exp. 2. ^c^ We only report results of Exp. 3.

Concerning the study of initiating joint attention with robot faces, a screen-based gaze-leading paradigm has been developed using a robot face instead of a virtual agent. In this gaze-contingent eye-tracking task with the face of the iCub humanoid robot (Metta, Sandini, Vernon, Natale, & Nori, 2008; Natale, Bartolozzi, Pucci, Wykowska, & Metta, 2017) presented on the screen, Willemse, Marchesi, and Wykowska (2018) manipulated the behavior of the robot to either follow the gaze of the participants (80% of the trials, “joint disposition” robot) or not (20% of the trials, “disjoint disposition” robot). In this way, authors could dissociate whether the modulation of re-engagement times to the faces arose from the learning of an agent’s identity (identity with disjoint disposition) or from trial-by-trial contingency. The results showed that onset times of saccades returning to the face of the robot were faster with the robot who typically followed the gaze than with the disjoint robot. Interestingly, the results extended previous findings and showed that this effect arose from the learnt disposition of the robot (main effect of disposition), and not by the trial-wise contingency (Willemse et al., 2018).

In this section, we observed that the majority of screen-based joint attention experiments using robots as attentional-orienting stimuli not only replicated classical findings of responding and initiating to joint attention but also essentially advanced our knowledge regarding the role of human-likeness in inducing joint attention mechanisms (Martini et al., 2015; Willemse et al., 2018). However, as argued above, screen-based agents might not be sufficient for elucidating social cognitive mechanisms.

### Joint attention examined with embodied robots and interactive protocols

Robots that are embodied and integrated into interactive protocols can act as dynamic social “partners,” which can engage mechanisms crucial for social cognition in daily life (Putman et al., 2006), see Fig. 1d. Being embodied, they increase social presence (Jung & Lee, 2004), and are more “natural” than even virtual reality, as they can modify our environment and manipulate physical objects around us. Importantly, they also allow for reciprocity in interaction: for example, similarly to virtual agents, robot’s gaze behavior can be programmed to be contingent on participants’ gaze. Moreover, similar to Gobel et al. (2015), one could exploit the dual function of robot gaze by manipulating participants’ beliefs about another human looking back at them through robot’s eyes. Finally, although it is still somewhat too early to have humanoid robots implemented in the “wild,” interactive paradigms in the lab that require joint actions and common goals with a human, such as manipulating objects on a table, could certainly have a real-life relevance, and are not constrained to tasks on the screens or 2-D environment. In the case of using humanoid robots in interactive scenarios, one can maintain experimental control while also embedding the setup in natural 3-D joint environment. Importantly for the purposes of studying joint attention, humanoids offer excellent experimental control—they can repeat same specific behaviors over many trials, and they allow for “modularity of control” (Sciutti, Ansuini, Becchio, & Sandini, 2015); that is, their movements can be decomposed into specific elements, an impossible endeavor for a human. For instance, in the context of joint attention research, the trajectory time of the movement of the eyes can be controlled and can follow predefined parameters over many repetitions. Overall, we argue that combining embodied humanoid robots with well-controlled experimental designs offers an optimal combination of ecological validity and experimental control, and allows for tapping into specific cognitive mechanisms such as joint attention.

A recent interactive study (Wykowska, Kajopoulos, Ramirez-Amaro, & Cheng, 2015) on joint attention involving an embodied robot iCub demonstrated that the gaze-cueing effect was of the same magnitude independent of whether participants believed iCub’s behavior was human-controlled or “programmed,” which is in slight contrast to previous studies with screen-based stimuli (Wiese et al., 2012). Similarly, Wiese, Weis, and Lofaro (2018) employing a gaze-cueing paradigm with Meka robot showed that the embodied robot elicited a gaze-cueing effect. Additionally, Kompatsiari, Perez-Osorio, et al. (2018) showed that the gaze-cueing effect during a gaze-cueing procedure with iCub humanoid robot was similar to those previously observed with human faces (Wykowska et al., 2014), at both the behavioral and neural level—that is, reaction times to target discrimination were faster, and the N1 ERP component peaked earlier and had higher amplitude on validly cued trials, relative to invalidly cued trials (Kompatsiari, Perez-Osorio, et al., 2018). Moreover, Kompatsiari and colleagues (2018) demonstrated that a real-time eye contact during a gaze-cueing paradigm with iCub enhances the gaze-cueing effect driven by a non-predictive cue (50% validity), while it suppresses orienting of attention driven by a counterpredictive gaze cue (25% validity), as compared to a prior no-eye-contact gaze. This paradigm, by encompassing an online eye contact prior to the gaze shift, challenges classical findings of screen-based paradigms that showed an automatic gaze-cueing effect elicited by counterpredictive cues (Driver et al., 1999; Friesen & Kingstone, 1998). Moreover, a similar nonpredictive gaze-cueing study showed that participants not only engaged in joint attention (measured by the gaze-cueing effect) merely when the robot established eye contact before shifting the gaze, but they also fixated longer on iCub’s face during eye contact than during no-eye-contact gaze (Kompatsiari, Ciardo, De Tommaso, & Wykowska, 2019a). These results advanced the knowledge related to the cognitive mechanisms affected by eye contact in joint attention research, by demonstrating that eye contact has a “freezing” effect on attentional focus, resulting in longer disengagement times and thus longer time to reallocate attention.

Besides being initiators of joint attention, humanoid robots can also be programmed to respond to the gaze of participants, thereby introducing reciprocity. In an interactive version of the screen-based gaze-contingent task, Willemse and Wykowska (2019) found an interactive effect of robot disposition (more likely to follow human gaze or more likely not to follow) and the effect of trial-wise contingency over re-engagement with the robot’s face (measured as onset latencies of return saccades to the robot face), thereby providing different pattern of results that 2-D screen-based stimuli. Similar to human–human studies in joint attention research, studies using embodied humanoid robots, also show that an embodied robot might produce a different pattern of results than screen-based stimuli (Kompatsiari,et al., 2018; Willemse & Wykowska, 2019).

To provide the reader with a clearer view of the results obtained in joint attention research using different kinds of setups (from classical to more naturalistic), we summarize in Table 1 the gaze-cueing studies that were reported in the previous sections. Table 1 shows that the effect size of validity varies not only across setups but also within the same setup. However, in the majority of the reported studies, the effect size lies in the range of a large effect (> .8), and in only a few studies the effect size is medium (.5–.8). Although the largest effect sizes are reported in the screen-based human/schematic setup, it should be noted that more interactive setups—that is, those including human or robot partners—still induce medium or large main validity effects. Moreover, it is also worth noting that the smaller effect size observed in a number of studies can be attributed to a low number of trials, or to the inclusion of a manipulation that reduced the strength of the main validity effect due to the lack of a validity effect in one of the conditions (e.g., Hietanen et al., 2006; Jones et al., 2010; Kompatsiari, et al., 2018; Kompatsiari, Perez-Osorio, et al., 2018; Martini et al., 2015).

### Limitations in using robots as stimuli to study joint attention

Although embodied robots in interactive protocols can lead to new insights regarding the joint attention mechanism, it is important to note that robots obviously cannot substitute a human interactive partner, or evoke exactly the same mechanisms as those involved in real-life spontaneous human–human interaction. However, this constraint is not exclusively related to the use of robots. It also applies in general to controlled experimental setups for studying social interactions (even between human agents), since the repetitive agent’s movements over a relatively long time period and the rather monotonous nature of the task cannot really represent a spontaneous interaction. Finally, even the knowledge of participants that they are under examination might modify their behavior. However, robot stimuli might have a specific limitation related to their artificial nature. It might be that, first of all, they might not be treated as a social entity (and therefore not evoke all possible mechanisms of social cognition) and second, they might evoke negative attitudes of some participants. This is particularly related to anxieties and fears that humans have toward robotic technology and artificial intelligence (Kaplan, 2004; Syrdal, Dautenhahn, Koay, & Walters, 2009). This issue could be addressed by measuring the bias toward robots (e.g., by qualitative measures) and applying statistical methods to control for effects of interindividual differences. Another potential constraint of using robots consists in possibilities of comparison between studies and generalizability of results since robots are often very different; and it is often the case that one lab works with only one specific robot, whereas another lab uses a different robot platform. To address this limitation, the comparison should be mainly performed within the same robotic platforms or using robots that could evoke similar gaze cues—that is, having similar mechanical characteristics of eyes.

However, despite the limitations, we argue that embodied robots embedded in interactive protocols that are grounded in well-established paradigms targeting specific mechanisms of social cognition can be extremely informative and serve the function of social “stimuli” of higher ecological validity than classical screen-based stimuli. Simultaneously, they allow for maintaining a high degree of experimental controlling contrast to human–human interaction protocols.

### General guidelines for using embodied robots in joint attention experimental protocols

From the results reviewed here, it emerges that embodied robots would benefit from complying with specific design properties for research and applications in the area of joint attention. In terms of appearance, robots probably need to have a moderate human-like appearance (60% human morph) as indicated by Martini and colleagues’ study, which showed that robotic agents with 100% robot-likeness or 100% human-likeness did not show a reflexive gaze-cueing effect (Martini et al., 2015). Additionally, despite the limitations regarding the implementation of biologically inspired robot eyes both in terms of cost and complexity, mechanical human-like eyes that can enable a gaze-cueing procedure are recommendable (for a review, see Admoni & Scassellati, 2017). It would also be beneficial if robots are endowed with algorithms that allow for the establishment of eye contact with participants since it has been shown that eye contact initiated by a humanoid robot increases perceived human-likeness and engagement with the robot (Kompatsiari, Ciardo, Tikhanoff, Metta, & Wykowska, 2019b). It also enhanced joint attention (Kompatsiari, et al., 2018). Furthermore, gaze contingency of robot behavior implemented in a more naturalistic setup (i.e., without eye-tracker) would benefit by embedding in robots algorithms that would allow for online detection of participant’s gaze and assessment of saccadic eye movement parameters. Finally, in order to ensure the reproducibility of the results and studies, authors should always report the controller used for producing robot’s movements, the desired kinematic parameters (e.g., eyes velocity), and the actual measured parameters.

## Application of joint attention studies in human–robot interaction in healthcare

In the previous sections, we discussed the new approach of using robots to investigate the mechanism of joint attention. This section will report studies in which fundamental research reaches out to application to healthcare.

Similar to neurotypical population, in clinical populations more natural settings are needed to achieve a good understanding of the mechanisms of social cognition (including joint attention). For example, individuals diagnosed with high-functioning autism are shown to experience impairments in the ability to use implicit social cognition mechanisms: they have difficulties in responding intuitively to socially relevant information during an online dynamic and fast-paced interaction with others (Schilbach et al., 2013). However, explicit social cognition mechanisms in offline experimental protocols often remain intact (Schilbach et al., 2013). Indeed, individuals diagnosed with high-functioning autism are reported to respond differently when they judge an interaction in the role of an observer, relative to being an actor: the role of observer enables participants diagnosed with high-functioning autism to take the time and think about the interaction, while having to take part of the interaction actively triggers their social impairments, as they experience an overwhelming amount of social information. Therefore, more naturalistic approaches are needed to fully understand the cognitive processes impaired in ASD.

Here, we focus on the use of robots in interactive protocols for individuals diagnosed with ASD. Because individuals diagnosed with ASD enjoy being engaged with mechanical and technological artifacts (Baron-Cohen, 2010; Hart, 2005)—due to the fact that these artifacts are less overwhelming (simplified design), less intimidating, and offer repetitive, predictable behaviors—it has been proposed that using robot during interventions could help therapists to train social skills in children diagnosed with ASD (Cabibihan, Javed, Ang, & Aljunied, 2013; Scassellati, Admoni, & Matarić, 2012; Wiese, Wykowska, & Müller, 2014).

Children diagnosed with ASD, among other social and cognitive deficits, show impaired initiation of joint attention (e.g., reduced use of common joint attention strategies, such as gestures, finger pointing, and grasping the hand of an adult) and diminished responsiveness to joint attention bids (American Psychiatric Association, 2013; Charman et al., 1997; Johnson, Myers, & American Academy of Pediatrics Council on Children With Disabilities, 2007; Mundy, 2018; Mundy & Newell, 2007). The impact of reduced engagement in joint attention in ASD may be far-reaching—by contributing to functional development of other mechanisms of social cognition (Mundy, 2018). Because training joint attention in children diagnosed with ASD showed positive effects on social learning and development (Johnson et al., 2007; Mundy & Newell, 2007), intervention approaches for increasing joint attention have been encouraged (Johnson et al., 2007).

**Fig. 2:**
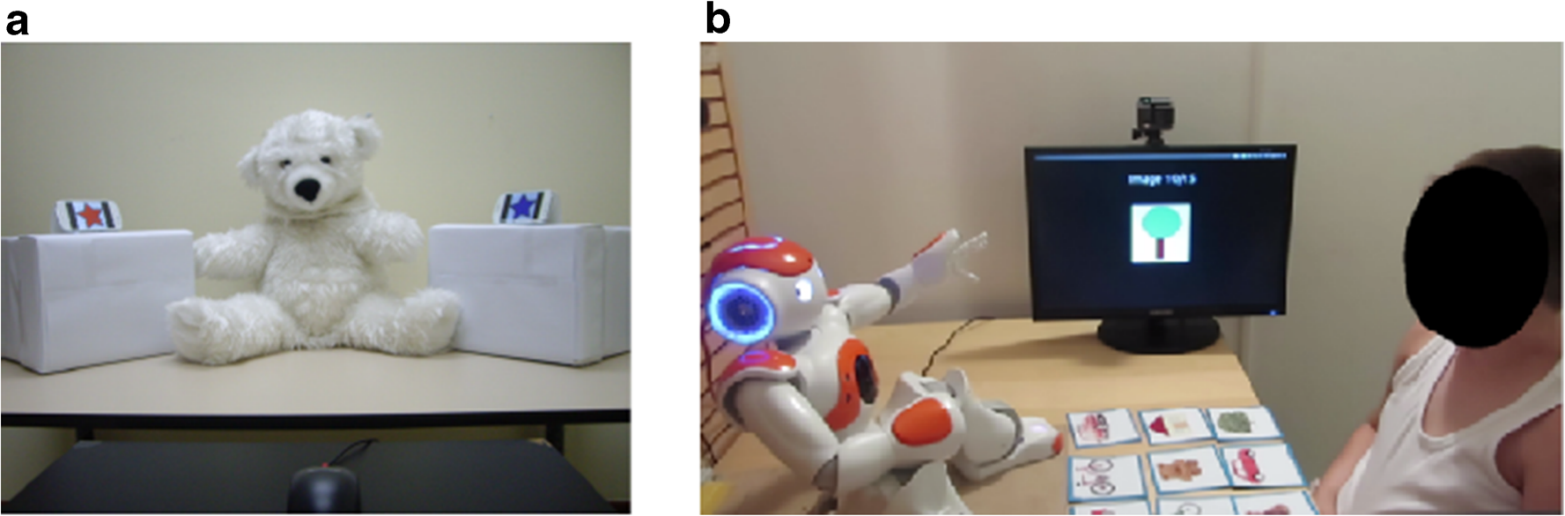
Examples of setups using robots to train and examine joint attention in children diagnosed with ASD. (a) Setup using the robot CuDDler. From “Robot-Assisted Training of Joint Attention Skills in Children Diagnosed With Autism,” by J. Kajopoulos et al., 2015, in A. Arvah, J.-J. Cabibihan, A. M. Howard, M. A. Salichs, and H. He (Eds.), *Social Robotics*, Cham, Switzerland: Springer. Copyright 2015 by Springer International Publishing Switzerland. (b) Setup using the robot Nao. From the thesis “Impact of Sensory Preferences in Individuals With Autism Spectrum Disorder on Their Social Interaction With a Robot,” by P. Chevalier, 2016, Université Paris-Saclay. Copyright 2016 by the author.

Following this line of reasoning, several authors focused on training or assessing the joint attention skills of children diagnosed with ASD with the use of interactive sessions with a robot (Anzalone et al., 2014; Anzalone et al., 2019; Bekele, Crittendon, Swanson, Sarkar, & Warren, 2014; Boccanfuso et al., 2017; Chevalier et al., 2017; David, Costescu, Matu, Szentagotai, & Dobrean, 2018; Duquette, Michaud, & Mercier, 2008; Kajopoulos et al., 2015; Michaud et al., 2007; Simut, Vanderfaeillie, Peca, Van de Perre, & Vanderborght, 2016; Taheri, Meghdari, Alemi, & Pouretemad, 2018; Warren et al., 2015; Zheng et al., 2013; Zheng et al., 2018), often through a *spatial attention-cueing paradigm*: The child is prompted by the robot to look in a given direction in which a visual target is displayed (see Fig. 2). The robots can use increasing degrees of bids for joint attention, depending on the child’s ability to respond to the bid (e.g., the robot will first move only the head, and if the child does not look at the target, the robot will prompt again by moving the head and pointing with the arm). However, using a robot for training or examining joint attention skills with individuals diagnosed with ASD was questioned by Pennisi et al. (2016): In their recent systematic review on autism and social robotics, they outline that results of studies on joint attention were mixed. Indeed, the five selected studies (published before November 3, 2014) on socially assistive robotics, focusing on joint attention in children diagnosed with autism, present contradictory and exploratory results. Anzalone et al. (2014) and Bekele et al. (2014) examined joint attention skills in children with ASD and typically developing children during a single interaction with a robot or a human partner. Both studies observed that a human partner needed less prompting (relative to a robot partner) to successfully orient the child’s attention. Duquette et al. (2008) and Michaud et al. (2007), however, observed higher improvements in the joint attention skills of two children diagnosed with ASD after training with a robot partner for 22 sessions, relative to two children diagnosed with ASD after training with a human partner for the same number of sessions. Finally, Warren et al. (2015) and Zheng et al. (2013) successfully trained joint attention skills in six children diagnosed with ASD with a four-sessions robot-based therapy, but they observed that the data obtained from their pilot study were not sufficient to suggest broader changes in the children’s skills. To summarize Pennisi et al. (2016) review, the benefits of a robot partner in comparison with a human partner to train and/or examine joint attention is not clear, however the studies are very exploratory considering the number of participants and their methodology (e.g., no pre- or posttest of the trained skills, single interaction, etc.).

In the following sections, we report and discuss more recent studies (published before July 15, 2018) evaluating the use of robot to train or examine joint attention in children diagnosed with ASD. Table 2 presents a summary of the articles reviewed here. Note, however, that the articles summarized in this review needed to satisfy two criteria: First, the studies reported in the articles needed to be human-centered (i.e., they were not focused only on the robotic system and skills). Second, their main purpose was to study the use of robots in therapy for children with ASD (i.e., the research needed to include clinical trials or scientific experiments, there needed to be at least an experimental group of children diagnosed with ASD, and the study needed to involve at least three participants diagnosed with ASD).

**Table 2:** Summary of the articles reviewed here

Study	*N*	Age	Gender (M–F)	Robot	Study Design	Control With a Human Partner	Measure Used	Main Results of the Study on Joint Attention
Anzalone et al., 2014^*^	16 ASD 14 TD	9.25 ASD 8.06 TD	13–5 ASD 9–6 TD	Nao	Cross-sectional with control (single session)	All participants interacted first with the human and then with robot partner	Task performance; Behavioral observations; 3-D motion tracking system	The robot needs higher level of prompting than the human partner
Anzalone et al., 2018 First study	25 ASD 12 TD	7.94 ASD 8.06 TD	10–5 ASD 10–6 TD	Nao	Cross-sectional without control (single session)	No	Behavioral metrics based on 3-D motion trackers	During joint attention with a robot, children with ASD and without ASD present different motion and gaze patterns
Second study	8 ASD	6.85	8–0	Nao	Cross-sectional without control (single session)	No	Behavioral metrics based on 3-D motion trackers	After training joint attention for 6 months (nonrobotic intervention), the use of the same setup of the first study enables to observe that the children’s motion and gaze behaviors are closer to typically developed children’s behaviors.
Bekele et al., 2013^*^	6 ASD 6 TD	4.7 ASD 4.4 TD	5–1 ASD 4–2 TD	Nao	Cross sectional with control (single session)	All participants interacted with the human and robot partners, with quasi-randomized order of presentation of the partner across participants	Task performance; Behavioral observations	Robot partner successfully induce joint attention in children with and without ASD; Robot partner needs higher level of prompting than a human partner
Boccanfuso et al., 2017	8 ASD	Between 3 and 6	?	CHARLIE	Longitudinal study (12 sessions)	Comparison between an experimental group (speech therapy + interaction with a robot) and a control group (speech therapy without robot)	Pre- and posttest with clinical questionnaires; Behavioral observations	Similar patterns in the children’s performances in joint attention if trained by a robot or by a human partner; Improvement of joint attention skills
Chevalier et al., 2016	11 ASD	11.9	9–2	Nao	Cross sectional without control (single session)	No	Task performance; Behavioral observations; Sensory profiles	Joint attention response time seems to be leaded by the sensory profiles of the participants
David et al., 2018	5 ASD	4.2	4–1	Nao	Longitudinal study (at least 16 sessions)	Each participant went through at least ~8 robot interventions and ~8 human interventions	Task performance; Behavioral observation	Similar patterns in the children’s behaviors and performances in joint attention if trained by a robot or by a human partner; Improvement of joint attention skills Robot partner needs higher level of prompting than a human partner
Kajopoulos et al., 2015	7 ASD	4.6	4–3	CuDDler	Longitudinal study (6 sessions)	No	Pre- and posttest with clinical questionnaire; Task performance	Improvements of the response to joint attention skills; Generalization of the trained skills from a robot to a human partner
Michaud et al., 2007^*^ Duquette et al., 2008^*^	4 ASD	5	3–1	Tito	Longitudinal study (22 sessions)	Two participants with a human partner, two participants with a robot partner	Task performance; Behavioral observation	Higher improvements in the joint attention skills after training with a robot partner than with a human partner
Simut et al., 2016	30 ASD	6.67	27–3	Probo	Cross sectional with control (single session)	All participants interacted with the human and robot partners, with randomized order of presentation of the partner across participants	Task performance; Behavioral observations	Similar patterns in the children’s performances in joint attention if trained by a robot or by a human partner; Improvement of joint attention skills
Taheri et al., 2018	6 ASD	8.67	6–0	Nao Alice-R50	Longitudinal study (12 sessions)	Yes	Pre- and posttest with clinical questionnaires; Behavioral observations; Task performance; Interview with subjects’ parents; Assessment of the participants pre- and postintervention by a clinical child psychologist	Too many games to be sure of the specific effects of the therapy on joint attention or imitation and the effect of the partner
Warren et al., 2013^*^ Zheng et al., 2013^*^	6 ASD	3.46	6–0	Nao	Longitudinal study (4 sessions)	No	Task performance; Behavioral observations	Improvement of joint attention skills
Zheng et al., 2018	14 ASD	2.78	12–2	Nao	Longitudinal study (4 sessions)	No	Task performance	Improvement of joint attention skills

For each study, we report the number of participants that effectively participated to the studies (*N*) and whether they were children diagnosed with autism spectrum disorder (ASD) or typically developed children (TD). ^*^These studies were included in Pennisi et al. (2016) systematic review.

### Robot-assisted training of joint attention in children diagnosed with ASD

Results from more recent studies using robots to train joint attention still report mixed results regarding the effectiveness of the method. For example, Simut et al. (2016) compared the behaviors of 30 children diagnosed with ASD during an interaction with a human or a robot partner, in a joint attention task. As in Anzalone et al. (2014) and Bekele et al. (2014), they observed no differences in the children’s performance in the joint attention tasks and in their behavior toward the different partners, except a longer gaze toward the robot partner. However, this is a single interaction, and no long-term effects could be observed. In a longer-term intervention, David et al. (2018), investigated if joint attention engagement of five children diagnosed with ASD was dependent on the social cues displayed by the robot during therapy sessions. They compared the effect of a human (~8 sessions) or a robot partner (~8 sessions) to train joint attention and compared the children’s performance in joint attention to their preintervention performance. As in Anzalone et al. (2014) and Bekele et al. (2014), they observed similar patterns in their five participants’ behaviors and performance in joint attention independent of whether the children were trained by a robot or by a human partner. Furthermore, the robot partner needed to show a higher level of prompting than the human partner. However, the study was performed including a small number of participants, and the joint attention skills were not evaluated posttraining, to assess the effectiveness of the therapy over a longer term.

Unlike the results of the previously discussed studies, Kajopoulos et al. (2015) found improvements in joint attention skills after a robot intervention. In their study, seven children diagnosed with ASD followed six joint attention training sessions with the robot CuDDler. Joint attention skills were evaluated before and after the training session, thanks to the abridged Early Social Communication Scale (ESCS; Seibert & Hogan, 1982). The ESCS enables to assess separately the mechanisms of responding to joint attention and initiating joint attention. The authors observed improvement in responding to joint attention, which is not surprising, given that the training protocol was designed to target specifically this mechanism with a head-cueing procedure. Importantly, however, improvement in responding to joint attention was observed during a human–human interaction session (the experimenter administering the ESCS posttest) two to three days after the end of the training. This is an encouraging result, showing that skills trained during human–robot interaction can be transferred to an interaction with a human. In Zheng et al. (2018), the authors presented an updated setup of their previous experiment from Bekele et al. (2014) and Zheng et al. (2013). In their earlier studies, the setup required a child to wear a hat and an experimenter to validate when the participant was looking at the target after the prompt of the robot (through a Wizard-of-Oz technique). In Zheng et al. (2018), the setup was automated and participants did not need to wear anything, which was a more convenient setup. The article describes the validation of their automated setup, with 14 children diagnosed with ASD that followed four sessions of joint attention training. They observed that during the sessions, the joint attention skills improved (the children looked significantly more to the target cue than to the nontarget cue across the sessions). However, as the authors point out, they did not use other screening tools to assess the improvements, and further studies should be conducted to replicate this result and examine whether the improvement transfers to interaction with human partners. In summary, although several researchers attempted a robot-assisted training of joint attention for children with ASD, the results remain mixed.

In addition to studies focusing only on joint attention in children diagnosed with ASD, other studies investigated robot-based set of games designed to train social skills, including, but not limited to, joint attention (Boccanfuso et al., 2017; Taheri et al., 2018). Boccanfuso et al. developed a low-cost robot, CHARLIE, to play a set of games designed to engage the children in imitation, joint attention, and social tasks. Over a period of six weeks, eight children diagnosed with ASD interacted with a robot partner in addition to speech therapy, whereas a control group of three children diagnosed with ASD participated only in the speech therapy. The children were screened pre and post-intervention with different screening tools, including the unstructured imitation assessment (UIA; Ingersoll & Lalonde, 2010). The UIA is a tool to measure a child’s ability to imitate spontaneously during unstructured play with an adult and has a subscale screening joint attention, which enabled the authors to track the children’s improvements in their joint attention skills. The authors observed that both groups benefited independently of the type of training, and the interaction with the robot did not provide additional benefits. In Taheri et al.’s study, the authors also developed a set of games involving imitation, joint attention, and social games with a robot. They compared the impact of a human partner and a robot partner for the improvements of the social skills of six children diagnosed with ASD that participated in the study. However, as the study involved only six children from different age groups, and the games were involving many skills, the authors reported that the results of their study could not give proper indication of the effect of the study on specific skills such as joint attention, or conclusions regarding the impact of human versus robot partners.

The results from these studies, despite being mixed, suggest that training joint attention with a robot improves the children’s joint attention skills, in a similar way as training with a human partner. However, this field of research requires more systematic and rigorous methods of testing and larger statistical power in the recruited samples, in order to validate the effects of socially assistive robotics in training joint attention.

### Examining the mechanisms of joint attention in children with ASD with robot interaction partners

Apart from *training* joint attention skills, robots can also be used as a tool to understand cognitive or behavioral mechanisms of joint attention in children diagnosed with ASD, or potentially, in the future, as a diagnostic tool. For example, in Kajopoulos et al.’s work (2015), in addition to training the mechanism of responding to joint attention bids, the authors used their experiment to observe the difference between the cognitive processes of responding to joint attention and initiating joint attention in children diagnosed with ASD. Because the children improved only in responding to joint attention bids, thanks to the spatial-cueing paradigm, this implies that both responding and initiating joint attention are different processes that are learned in a different way (as explained in Mundy, 2018). Their work also emphasized that robots can be used to target specific cognitive processes by using well-known paradigms used in laboratory settings that are designed to address isolated (in a controlled manner) cognitive mechanisms. Similarly, in Anzalone et al.’s work (2018), instead of using the robot for training joint attention skills, the robot was used to compare behavioral metrics of children with- and without a diagnosis of ASD performing a joint attention task. Furthermore, behavioral metrics of children with ASD were compared with the use of a robot before and after a period in which the children did the Gaming Open Library for Intervention in Autism at Home (GOLIAH; Bono et al., 2016). GOLIAH is a set of games (that does not involve robots) done in a clinic and at home that focus on training specific abilities, particularly joint attention and imitation. As in their previous work (Anzalone et al., 2014), the authors used the robot Nao in a gaze-cueing paradigm to assess a child’s response to joint attention. An RGB-D camera (Microsoft Kinect) was recording the gaze, body, and head behaviors during the experiment. The metric they used enabled statistical distinction of children diagnosed with ASD (*N* = 42) and without ASD (*N* = 16): Children diagnosed with ASD were less stable and their head and body moved more than neurotypical children during the joint attention interaction with the robot. This shows that the naturalistic interaction with the robot enabled measuring joint attention characteristics of children diagnosed with ASD and discriminating them from joint attention characteristics in typically developing children. The comparison of the behavioral metrics of eight children diagnosed with ASD before and after six months of training the joint attention skill thanks to GOLIAH showed that their body and head displacement and gaze behavior were closer to the pattern of typically developed children. In Chevalier et al. (2016), the authors used a spatial-cueing paradigm task to assess the different behavioral responses to a joint attention prompt from a robot partner regarding their participants’ sensory profiles. They hypothesized that the different sensory profiles in children diagnosed with ASD could lead to different behavior, and that assessing these interpersonal differences could help the knowledge of ASD and to better tune socially assistive robotics for this population. They assessed the sensory profiles of 11 children diagnosed with ASD and observed after a single intervention with a robot that the response time to joint attention from the robot seemed to be linked to the visual and proprioceptive preferences of the participants. However, the study was done only on a single session with few participants. Even if these results are obtained based on small groups of children and require replication, they are encouraging, and supporting the idea of the use of naturalistic robotic settings to *examine or diagnose* the mechanisms of cognitive process in children diagnosed with ASD.

### Limitations in the use of socially assistive robots for training and examining joint attention in ASD

The use of robots to train or examine joint attention skills in children diagnosed with ASD still provides inconclusive results, as discussed above. However, the field is still very new, and all the studies still have rather an exploratory, or proof-of-concept, character. Future research in training and examining joint attention with robots for children diagnosed with ASD should be conducted in a more systematic manner, with larger and well-screened samples, standardized pre- and posttests, appropriately designed control groups or conditions. Indeed, as Scassellati et al. (2012) explain in their review on research in socially assistive robotics for children diagnosed with ASD, research teams that develop these studies need to consist of experts specialized in many fields of research (to cite a few: robotics, computer science, psychology, etc.). Few research teams cover all these areas, and they tend to focus only on the strengths existing in that particular team. What is observed is that often, the experiments described are not targeted at specific isolated cognitive mechanisms. It is therefore difficult to observe and interpret precisely what changes during the therapeutic intervention. To explore social cognition mechanisms, although it is difficult to use exactly the same protocols as those developed for adults, it is still possible and recommended to adapt existing protocols in experimental psychology to children and to observe well-specified and isolated cognitive mechanisms.

ASD comprises of great inter-individual variability, as the symptoms fall on a continuum (American Psychiatric Association, 2013). Studies investigating the use of robots to train or examine joint attention in children with ASD rarely consider this aspect of ASD. However, as pointed out by Milne (2011), individuals diagnosed with ASD present very large interindividual differences, comparing to data collected from control groups. Furthermore, numerous studies used subgroups within their sample of individuals diagnosed with ASD to capitalize on the large differences in their symptoms and/or behaviors (Milne, 2011). The author also adds that although many cognitive deficits are observed in ASD, there are many studies in ASD literature with examples of not replicated results, suggesting that some of the observed specific cognitive impairments might not be consistent and universal in ASD. These observations from Milne can therefore also relate to the differences in results that have been observed in robot-assisted therapies of joint attention skills for children diagnosed with ASD.

It is also important to note that one major limitation of robot-based therapy reported in the studies discussed previously is on the technology used and the design of the training. Indeed, robot-based interventions aim to be more and more automated but are still limited in their range of actions due to technological limitations. Zheng et al. (2018) discuss that the design of the task used in their setup is limited by the automated system they developed and that in longer training protocols, the lack of different tasks could make the participants lose their interest and therefore make the therapy less impactful. Similarly, Anzalone et al. (2019) discussed that their automated setup offers limited freedom and that children find their behavior constrained. Chevalier et al. (2016) reported that they had to use a Wizard of Oz setup (i.e., the experimenter was controlling the robot instead of having an automated system). The face tracker technology they used during the experiment was unable to follow accurately the children’s faces as they covered their heads with their hands or they looked straight down, limiting the accuracy of the technology. Boccanfuso et al. (2017) used a teleoperated robot to test their games instead of the automated system they developed, to ensure that the robot was responsive rapidly and accurately enough to test more efficiently how engaging were the games they designed. The difficulty of designing robot-based interventions (quality of the games regarding difficulty, interest, etc.) is also pointed out in the previously discussed studies. David et al. (2018) reported that they had to change gradually the task to keep the children’s interest. Kajopoulos et al. (2015) and Chevalier et al. (2015) reported that even if the interventions were designed with the help of caregivers, some children had difficulties to understand or perform the task.

### Guidelines for robot-assisted training for ASD

As described above, the use of robots as a tool for training or examining joint attention skills in children diagnosed with ASD still yields mixed results. However, it should be noted that it is a promising avenue. Although it is a difficult process, progress might be achieved if future studies are based on closer collaboration with clinics, hospitals or associations working with children diagnosed with ASD. This should allow for the recruitment of a larger amount of participants over a longer time period. A larger number of participants could also mitigate the high prevalence of dropout rates or loss of data due to technical issues. Unfortunately, to date, too many articles report results on too few participants and/or for short-case studies, which makes it very difficult to draw conclusions regarding the results of the use of a robot in training children diagnosed with ASD. Additionally, working closely with clinicians should enable design of new training protocols with higher degree of engagement of participants in the training (see Chevalier et al., 2017; Ferrari, Robins, & Dautenhahn, 2009; Robins & Dautenhahn, 2010, for reports discussing design strategies for socially assistive robotic interventions). Another point for improvement is the evaluation of the children’s progress during training interventions. Using well-known paradigms or protocols is recommendable in order to target very specific cognitive mechanisms. For example, using the spatial attention-cueing paradigms, one can train responding to joint attention bids, but with the robot’s behavior being contingent on the gaze/head behavior of the participant (the robot following the gaze of a child), one can target the mechanism of initiating joint attention. Targeting one particular skill or set of skills, in a well-known structured way would ease the design of the experiments and the replicability of results and studies. Finally, using pre- and posttests to evaluate the progress of therapy and improvement in skills is also highly recommended. Finding appropriate clinical tests may be a challenge, depending on the country of study, as the ESCS, for example, is not translated in all languages. This is another reason to encourage close collaboration with clinicians. The above-mentioned guidelines should also help to take into account the great heterogeneity of the patients in ASD, which would enable to fit better the protocols and track more efficiently if certain subgroups of behavior exist in joint attention within the spectrum of autism. On a side note, open-source codes of the training intervention could additionally help in the replicability of studies.

## Conclusions and outstanding questions

In this review, we have discussed new approaches in examining joint attention, with a special focus on the use of embodied robots in healthy individuals and clinical population of individuals diagnosed with ASD. We highlighted that classical approaches with observational stance and screen-based stimuli do not capture all aspects of social cognition. Therefore, new approaches capitalizing on naturalistic and interactive setups (Schilbach et al., 2013) are more promising in terms of explaining various aspects of social cognition. However, using naturalistic approaches is challenging with respect to experimental control. In this context, humanoid robots can prove particularly useful, as they allow studying social cognition and joint attention specifically with both a high degree of experimental control and relatively high ecological validity. Such approach provides new insights into the mechanisms of joint attention (such as the role of human-likeness, and eye contact in eliciting gaze-cueing effects, and the difficulty in disengagement from the face during eye contact), and potential for application in healthcare, in training and examining joint attention in children diagnosed with ASD.

One crucial theoretical question that is not yet fully understood in joint attention research relates to how different nonverbal cues such as eyes, head, body posture or pointing are integrated in order to summon human’s attention. This question could be easily addressed with full-body humanoid robots that consist of mechanical eyes since the robot’s movements can be decomposed into individual components but also in selected combinations of them, as described in (Sciutti et al., 2015) by the term “modularity of the control.” The importance of this topic is also relevant for clinical studies. Previous research showed that in autism, a robot seemed to need a higher level of prompting than a human (e.g., a robot needed to use a combination of the face and arm whereas a human needed only the face, see Anzalone et al., 2014; Bekele et al., 2014; David et al., 2018). However, those studies did not examine the cognitive processes involved and the results are still very exploratory because of the small number of participants.

Similarly, the mechanical abilities of a humanoid robot could allow for exploring how the velocity of movements affects joint attention. This is also relevant for clinical studies in autism, as this population is known to have impaired processing of visual motion (Simmons et al., 2009). Some studies have observed that slowing down the velocity of videos would help children diagnosed with ASD in improving verbal cognition and behavior (Tardif, Latzko, Arciszewski, & Gepner, 2017), and in better exploration of facial signals (Charrier, Tardif, & Gepner, 2017).

The possibility of changing the appearance of robots, by modifying, adding, or removing elements of its body and face, could enable investigating how social and individual biases toward appearance can affect joint attention. Understanding the impact of appearance in joint attention could greatly help in clinical applications; for example, plain robotic faces and bodies have been discussed as being more efficient for interacting with children with autism than is more realistic, complex embodiment (Billard, Robins, Nadel, & Dautenhahn, 2007).

Another aspect of joint attention that could be thoroughly investigated using humanoid robots, but that is almost impossible to be examined with screen-based experiments, involves joint attention during joint action. This could theoretically boost joint attention research since the majority of dyadic or group interactions in real life are related to actions. The findings would directly help research in clinical studies target the processes impaired in interactions more efficiently.

### Author Note

This work is supported by the European Research Council (ERC) under the European Union’s Horizon 2020 research and innovation programme (grant awarded to A.W., titled “InStance: Intentional Stance for Social Attunement,” grant agreement no. 715058) and by Minded Program–Marie Skłodowska-Curie grant agreement no. 754490, a fellowship awarded to P.C.
